# POF Smart Carpet: A Multiplexed Polymer Optical Fiber-Embedded Smart Carpet for Gait Analysis

**DOI:** 10.3390/s19153356

**Published:** 2019-07-31

**Authors:** Leticia M. Avellar, Arnaldo G. Leal-Junior, Camilo A. R. Diaz, Carlos Marques, Anselmo Frizera

**Affiliations:** 1 Graduate Program in Electrical Engineering, Federal University of Espirito Santo, Vitoria 29075-910, Brazil; 2 Mechanical Engineering Department, Federal University of Espirito Santo, Espirito Santo 29075-910, Brazil; 3 I3N & Physics Department, University of Aveiro, Campus Universitário de Santiago, 3810-193 Aveiro, Portugal

**Keywords:** gait analysis, polymer optical fiber sensors, ground reaction force, spatio-temporal gait parameters, smart textile

## Abstract

This paper presents the development of a smart carpet based on polymer optical fiber (POF) for ground reaction force (GRF) and spatio-temporal gait parameter assessment. The proposed carpet has 20 intensity variation-based sensors on one fiber with two photodetectors for acquisition, each one for the response of 10 closer sensors. The used multiplexing technique is based on side-coupling between the light sources and POF lateral sections in which one light-emitting diode (LED) is activated at a time, sequentially. Three tests were performed, two for sensor characterization and one for validation of the smart carpet, where the first test consisted of the application of calibrated weights on the top of each sensor for force characterization. In the second test, the foot was positioned on predefined points distributed on the carpet, where a mean relative error of 2.9% was obtained. Results of the walking tests on the proposed POF-embedded smart carpet showed the possibility of estimating the GRF and spatio-temporal gait parameters (step and stride lengths, cadence, and stance duration). The obtained results make possible the identification of gait events (stance and swing phases) as well as the stance duration and double support periods. The proposed carpet is a low-cost and reliable tool for gait analysis in different applications.

## 1. Introduction

The human gait can be defined as a locomotion method characterized by periods of loading and unloading of the limbs with cyclical repetition [1]. Each gait cycle comprises of two steps and consists of the period from the initial contact of one foot to the following occurrence of the same event with the same foot [2]. The gait cycle can be divided in two gait phases, composed by stance (when the foot is on the ground) and swing phases [3]. These phases are subdivided into subphases, and the correct discrimination of these events can be considered the starting point for several scientific and clinical applications, such as the evaluation of gait in patients after interventions or rehabilitation treatments [4,5,6,7], the control of robotic devices for the recovery of lower limb mobility [8,9], distinguishing between normal and pathological gait [10,11].

Two main approaches are used for gait assessment: kinematics and kinetics. The kinematics studies the description of motion of the body without considering the causes of motion [12], such as joint angles, center of mass (CoM) displacement and velocity, and spatio-temporal gait parameters. The spatio-temporal gait parameters describe the gait relating the foot placement, gait events timing and velocity variables [3], and their measurement forms the basis of any gait analysis [1]. Among the technological devices commonly used for kinematic analysis of the human gait there are camera-based optical motion capture systems based on image processing, capable of collecting in three dimensions body joint positions and estimate spatio-temporal gait parameters and joint angles [13]. The marker-based optical motion capture systems are considered to be the gold standard for gait analysis and commonly used as reference [12]. Other solution for kinematic analysis are the inertial sensors, which are attached onto the surface of the human body, collecting the linear acceleration and angular velocities, allowing the combination of these data to estimate the joint angles and spatio-temporal gait parameters [2].

In contrast, kinetics studies the forces and torques that initiate the motion. It considers the forces generated internally in the body that result in human movement [12]. Kinetics parameters include ground reaction forces (GRF), plantar pressure distribution and joint momentum [14]. For kinetic analysis, the systems used are based on strain gauges, piezo-electric sensors, or force sensing resistors. Instrumented insoles are a portable solution for outdoor tests and can provide the foot plantar pressure distribution, through sensors located at specific points of the foot [15]. Besides instrumented insoles, there are force platforms, which collect the load and shear components of the GRF and center of pressure (CoP), using strain gauges or piezo-electric quartz crystals to convert force into electric signals [1]. However, the subjects must place their foot within the boundaries of the platform to avoid errors in measurement, which may alter their natural gait pattern, resulting in errors on the patient’s gait analysis [16].

As an emerging sensor technology, optical fiber sensors offer many advantages compared to traditional electronic sensors, such as lightweight, compactness, chemical stability, immunity to electromagnetic field and multiplexing capabilities [17]. In addition, their lower dimensions and flexibility enable the embedment in different materials [18] in order to create functional materials such as instrumented supports for wearable applications [19] and smart textiles [20,21].

Polymer optical fibers (POFs) present advantages over silica optical fibers related to their material features, such as higher flexibility, lower Young’s modulus, higher strain limits, fracture toughness and impact resistance [17]. There are many applications of POF-based sensors in clinical environment for gait analysis, such as sensor for monitoring joint angular motion [22], plantar pressure [23], gait cadence [24] and foot placement during the gait [25].

Different POF sensors approaches have been proposed throughout the years, such as intensity variation [26], nonlinear effects-based [27], fiber Bragg gratings (FBGs) [28] and interferometers [29]. Among those techniques, intensity variation-based sensors are the ones that present the lowest cost, highest simplicity in fabrication and in the signal processing [22]. As an important issue for intensity variation-based sensors, the lack of multiplexing capabilities inhibit the application of such sensors in multiparameters or multipoint measurement, since it will require one photodetector and one light source for each sensor [30]. To tackle this issue, a multiplexing technique based on side-coupling of the light source was proposed in [30], where the photodetectors are positioned on POF end facets and the optical power variation of the sensors are acquired by the photodetectors when each light source is active [30]. Such approach results in higher spatial resolution than techniques such as time-domain reflectometry and optical frequency-domain reflectometry (spatial resolution of some meters). In addition, the intensity variation-based multiplexing technique results in a system with lower cost than the ones based on Brillouin analysis and FBGs [30].

Aiming at these advantages, an instrumented insole for plantar pressure monitoring was proposed in [31] using the multiplexing technique for intensity variation-based sensors. The insole has 15 measurement points, which is the highest number of measurement points for an instrumented insole based on optical fiber sensors. Considering the advantages of the multiplexing technique as well as the possibility of embedded POFs in textiles, the purpose of this work is the development of a smart carpet with multiplexed POF sensors for kinematic and kinetic analysis of human gait. The collection of GRF data and spatio-temporal gait parameters was performed at 20 quasi-distributed points in a 2-m long smart textile. The proposed POF Smart Carpet is an interesting low-cost alternative with high scalability for gait analysis.

## 2. POF Smart Carpet Development and Experimental Procedures

**Figure 1:**
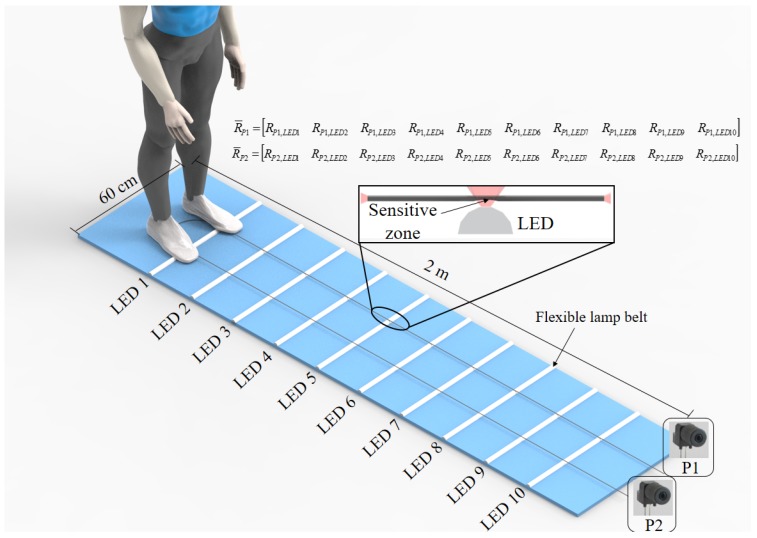
POF Smart Carpet overview.

The POF Smart Carpet structure is based on one POF arranged parallel to the walking direction in between two polyethylene layers, 60 cm wide and 2-m long (see Figure 1). The POF was made of polymethyl methacrylate, PMMA (HFBR-EUS100Z, Broadcom Limited) with a core diameter of 980 μm, a cladding of fluorinated polymer with 20 μm thickness and a polyethylene coating that results on a total diameter of 2.2 mm for the fiber considering its coating. The light source used is a light-emitting diode (LED) flexible lamp belt, horizontally arranged, and the sensors responses, i.e., the optical power variation, were acquired by the photodetectors IF-D92 (Industrial Fiber Optics, Tempe, AZ, USA). The signal acquisition and the LEDs control are performed by the microcontroller FRMD-KL25Z (NXP Semiconductors, Netherlands) and all the data were analyzed and processed in MATLAB (MathWorks Inc., Natick, MA, USA). The total estimated cost of the smart carpet is US$ 36.00, considering two polyethylene layers measuring 2 m (US$ 1.15 per meter), POF length of 4 m (US$ 0.80 per meter), two photodetectors (US$ 4.23 per photodetector), 2 m of flexible lamp belt (US$ 3.50 per meter) and microcontroller (US$ 15.00). This results in a lower cost system when compared with commercial systems usually used for gait analysis. It also should be noted that the proposed POF Smart Carpet has a modular configuration, i.e., the carpet can be assembled with different number of LED flexible lamp belts along the carpet and with different lengths.

The proposed smart carpet can measure the plantar pressure along the device and to estimate the spatio-temporal gait parameters using the sensors distributed throughout carpet. To enable the side-coupling of the light source and, at the same time, increase the sensor sensitivity, a lateral section is made on the fiber, where the cladding and part of the core are removed, creating the sensitive zones demonstrated in Figure 1 inset. The lateral section length, depth and surface roughness were made through abrasive removal of material following the guidelines presented in [32]. The sensitive zone is created using a sandpaper connected to a rotary tool. The POF is positioned on a fixed support where the rotary tool advancement is limited by another part over this support to guarantee the desired length and depth of the lateral section in the POF sensor [32].

In this case, 20 lateral sections were produced on the fiber with 20 cm separation from each other. Then, the fiber was turned in 180${}^{\circ }$ (as shown in Figure 1), which results in 2 rows with 10 sensors each, where there is a 15 cm horizontal separation between the rows. In addition, the LED flexible lamp belts are positioned on each lateral section. When the pressure is applied on each lateral section, there is an optical power variation, which is acquired by P1 and P2.

Two photodetectors were employed, one at each end of the fiber, to acquire the optical power variation of all 20 sensors $R_{LEDn}$. Thus, the responses of 10 sensors (${\bar{R}}_{P1}$) are acquired by P1 and the other 10 sensors (${\bar{R}}_{P2}$) are acquired by P2, as schematically demonstrated in Figure 1.

The multiplexing technique proposed by [30] comprises of a sequential activation of each LED with a predefined frequency and activation sequence. In this case, one LED flexible lamp belt is activated at a time, illuminating two sensors *n* simultaneously (right and left), where a microcontroller controls the activation frequency and sequence. The acquisition sequence is from LED 1 to 10 with an activation frequency of 30 Hz for each LED. In addition, the microcontroller is responsible for the acquisition of the optical power measured by each photodetector when each LED is active, resulting in two matrices, one for the P1 and the other for P2, as shown in Figure 1. In this case, each matrix has 10 columns, where the columns represent the optical power acquired by P1 and P2 when a predefined LED is active, and each row represents the temporal acquisition.

**Figure 2:**
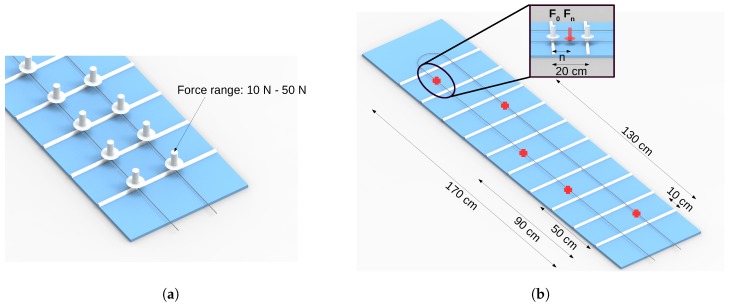
Characterizations protocols setup: (**a**) Force characterization. (**b**) Spatial characterization.

To perform the force and the spatial characterization of the carpet, two experimental protocols were applied. The first protocol is the force characterization, based on positioning of calibrated weights on top of each sensor on the range of 0 N to 50 N with steps of 10 N. The sensors responses are acquired by the photodetectors. Figure 2a shows the setup for force characterization. The second protocol is the spatial characterization, which is based on positioning of the foot on markers with predefined distances along the carpet. This protocol was performed by two volunteers. The goal of this protocol is to correlate the optical power with the distance along the carpet. This characterization is related to the force characterization, since it is necessary to obtain the sensor response with predefined weights exactly on top of the sensor. Figure 2b shows the spatial characterization setup.

The spatial characterization was based on continuous beam model [33], as shown in Figure 2b, in which the LED flexible lamp belts correspond to the supports and the fiber correspond to the beam. The optical power response for the force applied along the fiber in the region between two consecutive LED flexible lamp belts is inversely proportional to the distance of this force to LED flexible lamp belt. As the distance increases the optical power decreases, when compared to the case where the force is applied on the LED flexible lamp belt, which is the maximum optical power response (as shown in Figure 2a). Thus, we consider a linear variation of the force along the fiber with the maximum optical power variation when the force is applied in the region of the fiber on the lamp belt and the minimum optical power variation occurs on the region at the middle of two consecutive LED belts as presented in Figure 2b. The relationship between the optical power attenuation and the distance to the sensor was calculated by Equation (1), where $F_{0}$ is the force applied on top of the sensors, $F_{n}$ is the force applied along the fiber between the LED flexible lamp belts, *n* is the distance of $F_{1}$ to $F_{0}$ and *l* is the distance between the LEDs, equivalent to 20 cm.

(1) $$n=\frac{F_{n}\cdot l}{F_{0}}$$

Lastly, the third experimental protocol consisted of three walking tests, in which the volunteers started the tests with right foot. The goal of this protocol is to validate the previous characterizations and to estimate the GRF and spatio-temporal gait parameters during walks.

The spatio-temporal gait parameters analyzed in this work consist of the step and stride lengths, cadence, and stance time. Each heel strike is detected by first optical power variation of the sensor and the signals are analyzed in pairs with adjacent sensors. This method allows identification of the heel strike position based on the optical power variation of each sensor. Thus, the distance between the heel strike and the sensors (*n*) can be calculated by Equation (1). The distance of heel strike to carpet start position is the sum of the *n* with the position of LED flexible lamp belt related to the first stressed sensor in the walking direction. With each heel strike defined, it is possible to calculate the step length through the spatial difference between adjacent heel strikes. Furthermore, the sum of two steps results in the stride length. The cadence is equivalent to number of steps per minute. The time of each test is obtained by ratio between the total samples and activation frequency, while the number of steps is known through step length evaluation. Therefore, the stance time consists of the ratio between optical power variation time and activation time, since the sensor optical power variation only exist when the foot is on the ground pressing the fiber.

## 3. Results and Discussion

### 3.1. POF Smart Carpet Characterizations

**Figure 3:**
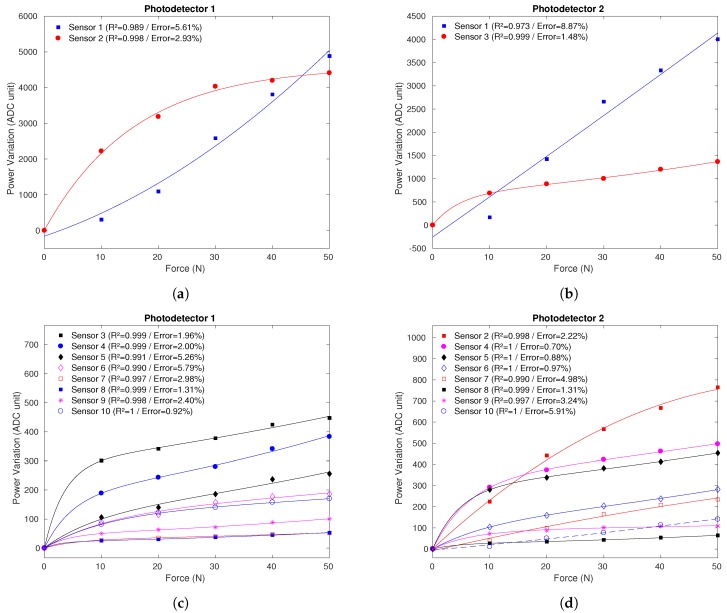
Sensor’s response in the force characterization with fitted curves: (**a**) Right sensors 1 and 2 using photodetector P1. (**b**) Left sensors 1 and 3 using photodetector P2. (**c**) Right sensors 3–10 using photodetector P1. (**d**) Left sensors 2 and 4–10 using photodetector P2.

Figure 3 presents the sensors response to the loads applied to 20 sensors (10 for each photodetector) in the force characterization, showing the determination coefficient (R${}^{2}$) and the relative errors, in which the markers represent the measured output and the continuous lines represent the sensors fit. It is noticeable in Figure 3c,d the exponential behavior of the curves obtained by P1 and P2, with a linear region from the application of the weights and a saturation tendency for higher weights. Figure 3a,b shows the response of the sensors with higher sensitivity than the other ones. The sensitivity and the linearity are related to the fabrication of each sensor, and for this reason the more sensitive sensors are different for each photodetector. The sensors 1 acquired by each photodetector present polynomial behavior, exceptionally.

**Figure 4:**
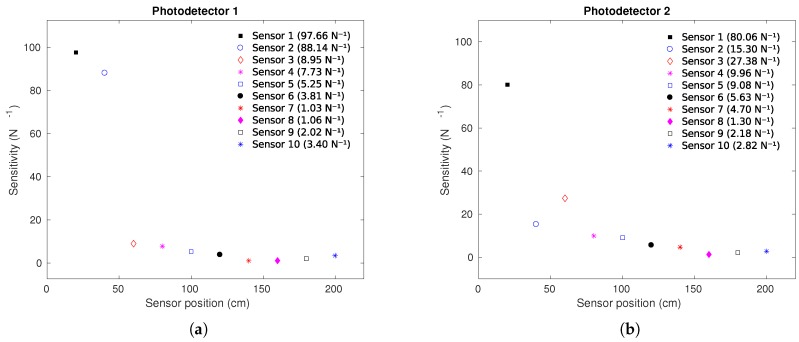
Sensitivity as a function of the sensor position: (**a**) Right sensors using photodetector P1. (**b**) Left sensors using photodetector P2.

Figure 4a,b presents the sensors’ sensitivities as a function of the sensor positions. It is possible to observe that generally, the sensitivity decreases as the distance between the sensors and the photodetectors increases. The reason of this behavior optical power attenuation at each lateral section leading to a lower detected optical power by the photodetector. This results in a lower sensitivity for the sensor with higher distance from the photodetectors. However, it is important to mention that the lateral section length and depth also influence the sensor sensitivity [32], which can explain the higher sensitivity of the sensor 3 acquired by P2 when compared with sensor 2 acquired by the same photodetector (P2).

**Figure 5:**
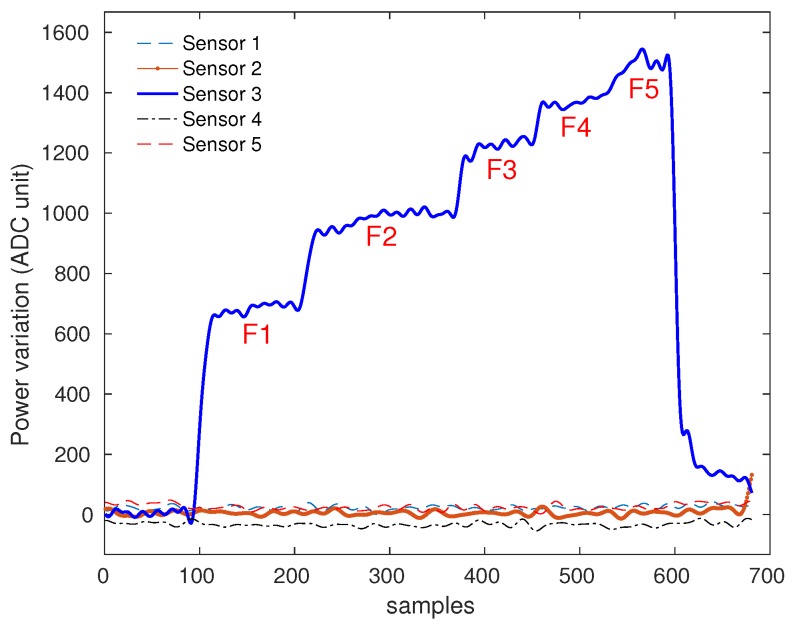
Response of sensors 1–5 when a loading is applied to sensor 3. The applied forces are of 10 N to 50 N, respectively, with steps of 10 N.

Figure 5 shows a significant difference between the response of sensor 3, where the force was applied, and the responses of the adjacent sensors (sensors 1, 2, 4, and 5). Considering the low power variation of the other sensors, the crosstalk between sensors is negligible. The cross-sensitivity between both photodetectors is only observed in sensor 10 due to the proximity of this sensor to photodetectors P1 and P2. However, human gait comprises of sequential contralateral steps [1], in which the foot will be placed in one fiber at a time. For this reason, we can identify if the sensor 10 variation was caused by the left or right region of the fiber by analyzing the previous steps. It is noticeable that the power variation decreases as the weight increases. This is due to the exponential behavior of the sensor with saturation tendency, showed in Figure 3.

**Figure 6:**
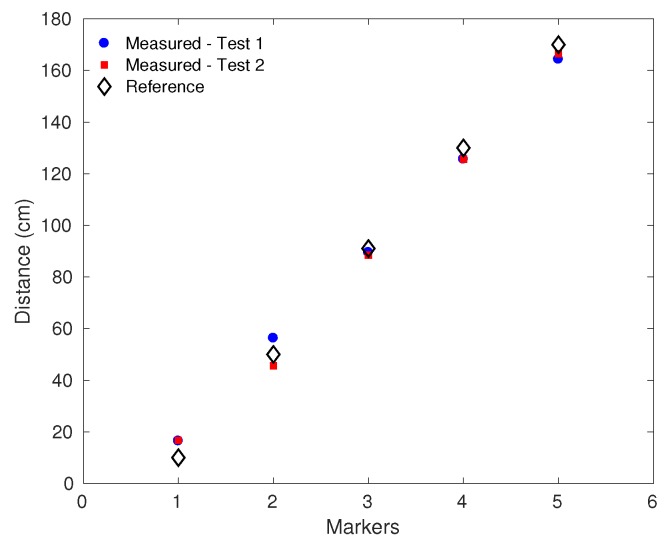
Results of spatial characterization in the two tests.

Two volunteers were asked to place their foot on predefined points of the carpet, which are defined in Figure 2b. Figure 6 shows the results of spatial characterization, where the maximum error of 6.7 cm was obtained in the marker 1 during the first test. The errors can be related to low sensitivity and/or nonlinearities of the sensors at the region of the first marker (sensors 9–10). On the other hand, the lowest error obtained was 1.5 cm, which occurred in the marker 3. The mean error on this characterization was 4.6±1.7 cm, which, considering the whole range of spatial characterization (160 cm), represents a relative error of 2.9%. The differences between tests 1 and 2 can be related not only to the sensor repeatability, but also to the minor differences on the foot positioning of the volunteers during the tests.

### 3.2. POF Smart Carpet Validation in Walking Test

**Figure 7:**
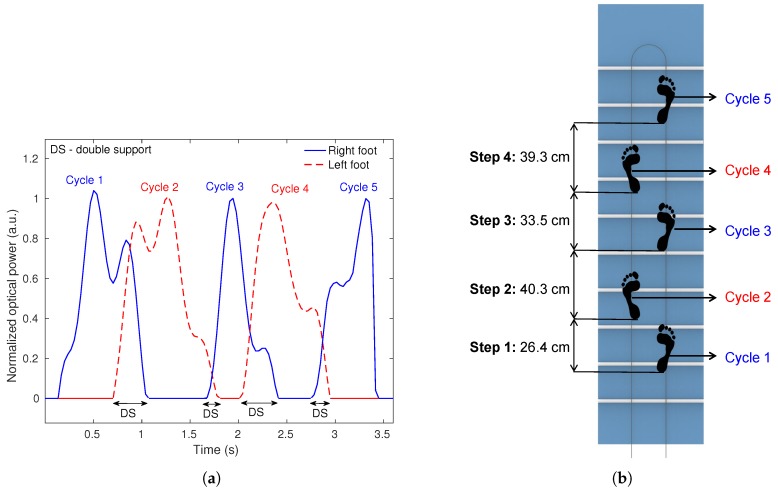
Results of one walking test: (**a**) Normalized GRF curves. (**b**) Foot placement including the step lengths.

To validate the POF Smart Carpet, three walking tests were performed by three volunteers (two males and one female), age of 26.3±2.1 years. The weight of each voluntary did not influence the results, since the sensor response was normalized for each weight and the purpose of this work is a qualitative analysis. The results of walking tests were divided into GRF data and spatio-temporal gait parameters, including step and stride lengths, cadence and stance duration, in % related to total walking duration. Figure 7a shows the normalized GRF curves of right and left foot and Figure 7b shows the foot placement including step lengths during one walking test. The GRF curves present similarity with normal M-shaped GRF pattern [1] and is possible to identify the gait events on the stance phase. It is noticeable that the curve pattern changes along the walking and can also be related to the type of foot strike, which can be neutral, pronated, or supinated. Furthermore, these deviations can also be related with lateral misplacement of the foot on the fiber leading to a variation on the stress transmission to the fiber resulting in different responses of the sensors, may distort the M-shaped. Nevertheless, it is still possible to identify the stance and swing phases, distinguishing the gait events on the stance phase, as well as the double support period in all analyzed cases.

Table 1 presents the results of spatio-temporal gait parameters. The volunteers were asked to perform four steps on each walking test. Since the tests were performed by young adults, the step and stride lengths were shorter than the ones commonly obtained in other gait experiments [3]. For walking tests applied to kids or older people, the step and stride lengths would be naturally shorter than the obtained results and can be analyzed with the same system due to the system modular configuration and high spatial resolution. The version of the POF Smart Carpet has 180 cm, which presumably results in a mean of step length of about 45 cm. It is worth noting that the step lengths presented in Table 1 generally are close to 45 cm, with a few deviations from this mean value due to the intrinsic variability of the gait [34]. The cadence variability can be related to self-selected pace, which results in a different velocity patterns for each test. However, it is possible to observe similarities on the cadence if the tests of each volunteers are analyzed (see Table 1).

Regarding to the stance duration, a mean and standard deviation of 63%±5% was obtained considering the total walking duration in all performed tests. It is important to mention that the normal stance duration during the gait is 60% according to the literature [1].

## 4. Conclusions

This paper presented the development of a smart carpet for kinetic and kinematic analysis through the monitoring of GRF and spatio-temporal gait parameters. The carpet comprises of low-cost intensity variation-based POF sensors with multiplexing technique based on side-coupling of the light source and can be an alternative to the high cost systems commonly used for gait analysis. Other advantage of the proposed system is the carpet length, which enables a gait analysis with more steps, with additional possibility of scalability, i.e., a longer carpet can be designed according to the necessities of the user. For this reason, this feature enables the approximation between the gait analysis tests and the normal gait. Results of characterizations showed exponential behavior of the sensors with a linear region from the application of the weights and a saturation tendency for higher weights, with high correlation coefficients. Furthermore, the results of the spatial characterization presented low errors through the proposed impact detection technique. Results showed that GRF curves are similar with literature [1], making it possible to identify gait events, stance duration, and double support periods. In addition, it was possible to estimate the step and stride lengths as well as the cadence. Future works include the addition of fibers with parallel configuration resulting in more rows for lateral displacement sensing to improve the reliability and making possible the step width estimation.

## Figures and Tables

**Table 1:** Spatio-temporal gait parameters in walking tests.

		Voluntary 1			Voluntary 2			Voluntary 3		
		Test 1	Test 2	Test 3	Test 1	Test 2	Test 3	Test 1	Test 2	Test 3
Step length (cm)	Step 1	26.4	42.9	16.5	46.5	35.2	30.3	56.5	39.5	38
	Step 2	40.3	31.9	56.9	35.5	35.6	51.1	31.0	35.2	31.1
	Step 3	33.5	42.1	48.1	27.0	39.0	34.7	40.6	55.0	56.4
	Step 4	39.3	37.1	27.2	35.7	34.8	27.8	31.7	10.7	14.9
Stride length (cm)	Stride 1	66.7	74.9	73.5	82.0	70.8	81.4	87.5	74.7	69.1
	Stride 2	73.8	74.1	105.0	62.5	74.6	85.8	71.6	90.2	87.5
	Stride 3	72.9	79.3	75.3	62.7	73.8	62.6	72.3	65.7	71.3
Cadence (steps/min)	-	81.8	62.5	63.4	44.3	44.8	46.9	59.6	60.0	65.7
Stance duration (%)	-	60.9%	69.4%	58.0%	54.3%	60.2%	65.4%	65.8%	68.5%	64.9%
